# Usefulness of the Bolus-Tracking Baseline Scan for the Diagnosis of Hepatic Steatosis in Abdominal Computed Tomography: A Feasibility Study

**DOI:** 10.5402/2013/147632

**Published:** 2013-04-27

**Authors:** J. Gossner, S. Schäfer

**Affiliations:** ^1^Department of Clinical Radiology, Evangelisches Krankenhaus Göttingen-Weende, An der Lutter 24, 37074 Göttingen, Germany; ^2^Radiology Department, Diagnostisches Zentrum Göttingen, Nikolausberger Weg 41 a, 37073 Göttingen, Germany

## Abstract

Nonalcoholic fatty liver disease (NAFLD) is a common pathology in western societies. Unenhanced computed tomography (CT) of the liver is a valuable tool in determining the presence of steatosis hepatis, but in most departments standard CT protocols of abdomen often do not include unenhanced scans anymore. In a small series of 22 patients the liver density was measured in the acquired low-dose baseline scan for bolus tracking and was compared to the measurement in a regular unenhanced CT scan of the upper abdomen. The mean difference between the unenhanced CT scan and the low-dose baseline scan was 3.4 HU (range 0.2–8.6 HU); the difference between these two scans was 5 HU or smaller in 82% of the patients. There was a significant difference between the two used CT scanners; this has to be kept in mind before implementing this approach into daily practice. All but one patient with fatty liver disease on unenhanced CT were diagnosed using the baseline scan. The baseline scan for bolus tracking may be useful for the diagnosis or in the followup of fatty liver disease.

## 1. Introduction

Nonalcoholic fatty liver disease (NAFLD) is common in western societies and is reported to occur in 10–24% of the general population. Once believed to be a benign condition, it has been shown that the occurring inflammatory changes may lead to steatohepatitis, cirrhosis, liver failure, and hepatocellular carcinoma [1]. Unenhanced computed tomography (CT) of the liver is a valuable tool to determine the presence and to quantifiy the extent of steatosis hepatis. After the administration of contrast media the assessment of liver fat contant is problematic, and only severe cases of NAFLD may be recognised [2, 3]. In most departments standard CT protocols of abdomen often do not include unenhanced scans of the liver anymore; this is hindering the diagnosis of fatty liver disease. Modern CT scanners are using bolus tracking for the optimal timing of contrast media application. The baseline scan for this bolus-tracking technique is an unenhanced low-dose axial scan. If these planning images can be used to determine the density of the liver parenchyma has been studied in a small series of patients.

## 2. Material and Methods

Retrospective Review of CT Datasets of 22 Patients. Patients were included if they had a multiphasic abdominal CT scan including unenhanced images of the upper abdomen and if the planning scan for bolus tracking included parts of the liver. Patients with focal liver disease were excluded. Patients were examined using a 16-slice CT scanner (Activion, Toshiba Medical Systems, Tokio, Japan) as well as a 64-slice CT scanner (Somatom AS, Siemens Medical Systems, Erlangen, Germany). The planning scan for bolus tracking consisted of a single axial unenhanced image acquired with low dose technique (Toshiba SureStart, Siemens SmartPrep). The unenhanced scans were performed with the use of automatic exposure control and a slice thickness of 1 mm. Datasets were postprocessed on a standard medical workstation (ReportDirect, Toshiba Medical Systems, Tokio, Japan). Mean attenuation values (Hounsfield units, HU) in comparable, representative, and homogeneous areas of the liver were obtained using regions of interest. The unenhanced CT scan of the liver was defined as the standard and compared to the baseline scan for bolus tracking. Descriptive statistics were performed. The mean differences between the unenhanced scan and the baseline scan of the two used CT scanners were compared using the Mann-Whitney *U* test.

## 3. Results

The mean difference between the unenhanced CT scan and the low-dose baseline scan was 3.4 HU (range 0.2–8.6 HU). In 82% of the patients the difference was 5 HU or smaller. Using the 16-slice CT scanner (Toshiba Activion) the mean difference was 4,6 HU (range 0.2–8.6 HU), and using the 64-slice CT scanner (Siemens Somatom AS) the mean difference was 2.1 HU (range 0.7–4.9 HU). The complete data is shown in Table 1. The mean differences between the two scanners were significantly different (*P* < 0.05). The 64-slice CT scanner was more accurate than the 16-slice CT scanner. Setting a threshold of 40 HU for the diagnosis of fatty liver disease, 3 out of 4 patients were positive also on the on the low-dose baseline scan (75%). The standard deviation was larger in the low-dose baseline scan compared to the unenhanced scan. The estimated effective dose of the baseline scan was around 0.05 mSv.

## 4. Discussion

**Figure 1:**
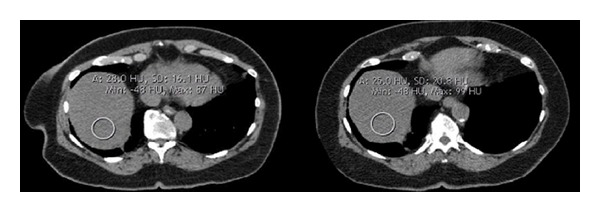
Measurement of liver density in a 72-year-old patient showing density lower than 40 HU suggestive of steatosis hepatis. The measurement on the low-dose baseline scan for bolus tracking (25 HU, on the right) is only slightly different compared to the unenhanced CT scan (28 HU, on the left).

The measurement of liver density on unenhanced CT scans has been shown to be one of the most reliable methods to determine fatty liver disease [2, 4]. The gold standard for the determination of liver fat is histology, but given its invasive nature it is not used routinely in patients with NAFLD. Despite newer techniques, for example ultrasound and magnetic resonance elastography or magnetic resonance spectroscopy, unenhanced CT scans of the liver are fast and easy to perform on every CT scanner without dedicated software. The density measurement of HU is an objective approach allowing comparison on followup examinations. A density below 40 HU on unenhanced CT scans is generally believed to be highly predictive of moderate to severe fatty liver disease and also identifies patients with the highest risk of disease progression [1, 2, 4, 5]. The measurement of liver density after the administration of contrast media is complex and not easily reproducible; this is mainly due to the use of different protocols for contrast-enhanced CT scans in different departments [3]. In most departments unenhanced CT scans are no longer part of every abdominal CT protocol. This does not constrain major diagnosis like malignancy or inflammation, but the diagnosis of fatty liver disease is hindered [6]. According to our data the use of the baseline scan for bolus tracking may help to solve this dilemma. Density measurements on these unenhanced low-dose images are within acceptable limits to make the suggestion of a possible pathological liver fat content, and further diagnostic may be advised (Figure 1).

The differences in density measurements can be explained by the acquisition of the planning images with low-dose and therefore elevated image noise; this is reflected by the larger standard deviation of the HU measurements in the baseline scans. Because of these found differences in liver density, measurement around the threshold of 40 HU should be interpreted with caution, and in these cases formal unenhanced CT scanning should be advocated. The 64-slice CT scanner was more accurate than the 16-slice CT scanner (*P* < 0.05). These differences have to be kept in mind; that means, before implementing this approach in daily practice the accuracy of the used scanner should be evaluated. 

The possibility to identify fatty liver disease with a low-dose CT scan has been shown by Boyce et al. [5]. But, until now, there is no data directly comparing unenhanced CT of the upper abdomen with low and standard dose regarding the diagnosis of steatosis hepatis. Our data has shown that differences are within acceptable limits for the diagnosis of fatty liver disease. But more reasearch on this topic is needed. 

The strength of our approach is that it uses already existing images; that is, there is no additional radiation exposure. The estimated radiation exposure for the baseline scan is 0.05 mSv; this equates to the dose of a chest radiograph. This makes followup examinations for the objective quantification of changes in liver fat content possible even in younger adults without applying concerning amounts of radiation. Our approach is also quick and easy to perform, which is important with ever-increasing workload. A major drawback of this approach is the possibility that focal fatty disease can be easily missed. The main limitation of our series is the retrospective design and the small sample size. So the reported data is very preliminary and needs to be replicated in a larger and prospective study. 

In conclusion liver density measurement of the baseline scan for bolus tracking may help to suggest steatosis hepatis in patients undergoing contrast-enhanced CT of the abdomen. 

## Figures and Tables

**Table 1:** Complete data of the patient series.

Patient	Scanner	Liver density on unenhanced CT (HU)	Liver density on baseline scan (HU)	Difference (HU)
1	Activion	54.6	53.6	1
2	Activion	50.8	45.8	5
3	Somatom AS	57.5	58.2	0.7
4	Activion	51.4	50.0	1.4
5	Somatom AS	53.5	58.2	4.9
6	Activion	28.0	25.0	3
7	Somatom AS	61.4	62.9	1.5
8	Somatom AS	61.1	60.8	0.3
9	Activion	55.1	50.7	4.6
10	Activion	49.8	43	6.8
11	Somatom AS	55.1	52.1	3
12	Somatom AS	56.5	58.2	1.7
13	Activion	50.3	44	6.3
14	Activion	52.5	52.3	0.2
15	Somatom AS	63.8	62.7	1.1
16	Somatom AS	58.6	61.4	2.8
17	Activion	58.5	51.7	6.7
18	Activion	60.7	69.3	8.6
19	Activion	20.3	24.5	4.2
20	Activion	36.3	33.2	3.1
21	Somatom AS	37.4	41.4	4
22	Somatom AS	56.2	59.3	3.1
